# Dependence of Quantum Dot Toxicity In Vitro on Their Size, Chemical Composition, and Surface Charge

**DOI:** 10.3390/nano12162734

**Published:** 2022-08-09

**Authors:** Alyona Sukhanova, Svetlana Bozrova, Evgeniia Gerasimovich, Maria Baryshnikova, Zinaida Sokolova, Pavel Samokhvalov, Chris Guhrenz, Nikolai Gaponik, Alexander Karaulov, Igor Nabiev

**Affiliations:** 1 Laboratoire de Recherche en Nanosciences, LRN-EA4682, Université de Reims Champagne-Ardenne, 51100 Reims, France; 2 Laboratory of Nano-Bioengineering, National Research Nuclear University MEPhI (Moscow Engineering Physics Institute), 115409 Moscow, Russia; 3 Laboratory of Experimental Diagnostics and Biotherapy of Tumors, N.N. Blokhin National Medical Research Center of Oncology, Ministry of Health of Russian Federation, 115478 Moscow, Russia; 4 Physical Chemistry, Technische Universität Dresden, Zellescher Weg 19, 01069 Dresden, Germany; 5 Department of Clinical Immunology and Allergology, Institute of Molecular Medicine, Sechenov First Moscow State Medical University (Sechenov University), 119146 Moscow, Russia

**Keywords:** quantum dots, semiconductor nanocrystals, cytotoxicity, nanotoxicity, nanomaterial biological interactions

## Abstract

Semiconductor nanocrystals known as quantum dots (QDs) are of great interest for researchers and have potential use in various applications in biomedicine, such as in vitro diagnostics, molecular tracking, in vivo imaging, and drug delivery. Systematic analysis of potential hazardous effects of QDs is necessary to ensure their safe use. In this study, we obtained water-soluble core/shell QDs differing in size, surface charge, and chemical composition of the core. All the synthesized QDs were modified with polyethylene glycol derivatives to obtain outer organic shells protecting them from degradation. The physical and chemical parameters were fully characterized. In vitro cytotoxicity of the QDs was estimated in both normal and tumor cell lines. We demonstrated that QDs with the smallest size had the highest in vitro cytotoxicity. The most toxic QDs were characterized by a low negative surface charge, while positively charged QDs were less cytotoxic, and QDs with a greater negative charge were the least toxic. In contrast, the chemical composition of the QD core did not noticeably affect the cytotoxicity in vitro. This study provides a better understanding of the influence of the QD parameters on their cytotoxicity and can be used to improve the design of QDs.

## 1. Introduction

The development of integrated pharmaceutical agents that could be used in both diagnosis and personalized targeted treatment is a prevailing trend in modern medicine. A common approach to this issue is the use of nanomaterials as components of preparations that are essentially combinations of active pharmaceutical agents and multifunctional nanoparticles with various structures. Quantum dots (QDs), fluorescent nanocrystals up to 10 nm in size, are the most widely used and extensively studied nanomaterials. They are most commonly used as fluorescent agents for highly sensitive in vivo imaging [1] or in vitro diagnostics [2] as well as in surgical manipulations, but they can also serve as drugs themselves, e.g., in photodynamic therapy [3]. Apart from the only clinical trial on the diagnosis of melanoma with the use of QDs approved in 2011 [4], to the best of our knowledge, QDs have not been used in clinical practice because of the numerous issues and contradictions concerning their toxicity [5].

The specific mechanisms of nanoparticle toxicity have been studied in sufficient detail. Cd-containing QDs have been found to cause DNA damage [6,7] and cytoskeletal alterations [8]. A number of studies evidence that QDs may induce excessive production of ROSs (reactive oxygen species) which may oxidize proteins, membrane lipids, and nucleic acids and, conclusively, cause mitochondrial dysfunction [9]. CdTe QDs have been shown to induce apoptosis [10], while both CdTe and CdTe/CdS/ZnS QDs decrease the viability of cells via an autophagy-dependent mechanism [11]. In contrast, CuInS_2_/ZnS QDs have a low cytotoxicity and cause no significant increase in ROS production [12].

It should be noted that the cytotoxic effects depend not only on external factors, such as the cell or body location of QDs and the local biological environment, but also on the properties of the QDs themselves, including the chemical composition, size, shape, and the chemical and physical characteristics of their inorganic and organic outer shells, such as the surface charge and hydrophilicity/hydrophobicity, as well as the resistance to environmental factors [13]. Given the QD structure, their toxicity should be considered as a function of the chemical compositions of the core and inorganic shell, the QD size, and the physical and chemical characteristics of the organic outer shell determining the QD properties in biological media and the mechanisms of QD interaction with biological molecules and structures. It is supposed that the contribution of different factors to the overall cytotoxicity can be ranked as follows: charge > functionalization > size [14]. The analysis of nanoparticle toxicity at the cellular and molecular levels is necessary not only for revealing the possible harmful consequences of the use of nanoparticles but also for finding ways to reduce their toxicity.

In this study, we compared the cytotoxicity of QDs with different chemical compositions, sizes, and the properties of the organic outer shell in two in vitro models in order to analyze the effect of each factor in more detail.

## 2. Materials and Methods

### 2.1. Synthesis of Quantum Dots

Core/multishell CdSe/CdS QDs (with eight monolayer (8 ML) of CdS) and CdSe/CdS/ZnS QDs (with 6 ML of CdS and 3 ML of ZnS) were synthesized by the method of continuous shell precursor injection described in more detail in [15]. The synthesis of CdSe/ZnS (3 ML, hereafter denoted by CdSe/ZnS) QDs was performed according to the protocol reported in [16]. CuInS_2_/ZnS QDs were obtained by colloidal synthesis in organic medium by a two-stage shell growth procedure as described in [17]. Finally, PbS/CdS/ZnS QDs were obtained in a sequential synthetic routine starting from hot-injection fabrication of PbS cores and their treatment by ion exchange as reported in [18] and later overcoating of the intermediate thin-shell PbS/CdS QDs by a thick two-component CdS/ZnS shell.

### 2.2. Optical Characterization of Quantum Dots

The absorption spectra of CdSe/ZnS, CdSe/CdS/ZnS (6+3 ML), and CdSe/CdS (8 ML) QDs were measured using a Cary 60 UV-Vis spectrophotometer (Agilent, Santa Clara, CA, USA). The fluorescence excitation and emission spectra of these QDs, as well as CuInS_2_/ZnS QDs, were measured using a Varian Cary Eclipse spectrofluorometer (Agilent, Santa Clara, CA, USA). An AvaSpec-NIR256-1.7 spectrophotometer (Avantes, Apeldoorn, Netherlands) was used for the characterization of PbS/CdS/ZnS QDs.

### 2.3. Transmission Electron Microscopy

Transmission electron microscopy (TEM) measurements of the QDs were performed using a JEOL JEM-1400Plus microscope (Croissy Sur Seine, France) operating at 120 kV. The TEM samples were prepared by dropping 10 μL of a diluted QD solution onto a Formvar/carbon coated 200 mesh copper grid and subsequently evaporating the solvent under ambient conditions.

### 2.4. Obtaining Water-Soluble Cdse/Zns (3 ML), Cdse/Cds/Zns (6+3 ML), Cdse/Cds (8 ML), Cuins_2_/Zns, and Pbs/Cds/Zns Quantum Dots

For obtaining water-soluble QDs, a 20 mg sample of QDs of each type was placed into a 2 mL test tube (Eppendorf France SAS, Montesson, France), and 800 μL of chloroform (Sigma-Aldrich, Saint-Quentin-Fallavier, France) was added. For purifying the original organically QD solution was intensely stirred in a test tube for 2 min. Afterwards, 1200 μL of methanol (Sigma-Aldrich, Saint-Quentin-Fallavier, France) was added to the QD solution in chloroform. The mixture was carefully stirred on a shaker and centrifuged at room temperature at 14,000 rpm for 5 min. After the centrifugation, the supernatant was removed, and the QD pellet was resuspended in 800 μL of chloroform. The cycle of washing, including QD dissolution in chloroform, addition of methanol, centrifugation, and withdrawal of the supernatant, was repeated three times. After the third centrifugation, The QD pellet was dissolved in 800 μL of chloroform by intensely stirring. Then, a 10 mg/mL DL-cysteine (Sigma-Aldrich, Saint-Quentin-Fallavier, France) solution in methanol was prepared. 200 μL of the DL-cysteine solution was added to the chloroform solution of every type of QDs under constant stirring. The resultant mixture was centrifuged at room temperature at 14,000 rpm for 5 min. After the centrifugation, the supernatant was withdrawn to remove unbound DL-cysteine. 1000 μL of methanol was added to the QD pellet and unbound DL-cysteine was washed off three times by centrifugation at 14,000 rpm for 3 min, which was followed by withdrawal of the supernatant, and another addition of methanol. After the last washing step, the supernatant was removed, and the QD pellet was dried in a Concentrator Plus vacuum concentrator for 2 min at room temperature to eliminate the remaining methanol. Then, 600 μL of water and 50 μL of 1 M sodium hydroxide were added to the dry residue of QDs. The resultant mixture was intensely stirred to dissolve the QDs in water. For better dissolution, the QD samples were placed onto an ultrasound water bath for 20 min. After that, the QD solutions were centrifuged at 8000 rpm for 10 min at room temperature. The QD solution obtained after centrifugation was filtered through a 0.22 μm Millex Syringe-driven Filter Unit (Sigma-Aldrich, Saint-Quentin-Fallavier, France).

To calculate the mass concentrations of the QD preparations when studying the effect of size and surface charge on QD toxicity, the absorption spectra of CdSe/ZnS, CdSe/CdS/ZnS (6+3 ML), and CdSe/CdS (8 ML) QD solutions were recorded, and the QD concentrations were calculated from the estimated first-exciton optical densities using the Beer–Lambert–Bouguer law, with the calculated molar weight of QDs and the QD sample dilution factor taken into account.

The concentrations of CdSe/ZnS QD or the infrared CuInS_2_/ZnS and PbS/CdS/ZnS QDs were calculated by the weight method when studying the effect of chemical composition on QD toxicity. After the final purification of the QDs, 35 μL of the QD solution was placed into a preliminarily weighted 0.5-mL low-bind test tube (Eppendorf France SAS, Montesson, France) and then dried in a Concentrator Plus (Eppendorf France SAS, Montesson, France) for 3 h at the temperature of 30°C. After that, the test tube was weighted again. The quantity of QDs contained in 35 μL of the original QD solution was calculated by subtracting the initial weight of the empty test tube from the final weight of the test tube containing the QD preparation after drying. The QD quantity per milliliter of solution was calculated to obtain the mass concentration.

### 2.5. Modification of the Quantum Dot Surface with Polyethylene Glycol Derivatives

When the QD concentration in the solution had been determined, the amount of organic ligands based on thiol-containing polyethylene glycol (PEG) derivatives that is necessary for the QD surface modification was calculated. Generally, three PEG derivatives were used: HS-(CH_2_)_11_-EG_6_-OH, HS-(CH_2_)_11_-EG_6_-OCH_2_-COOH, and HS-(CH_2_)_1_1-EG_6_-NH_2_ (ProChimia Surfaces, Gdynia, Poland). For the modification of all types of QDs, mixtures of QDs at the following ratios were used: 70% of HS-(CH_2_)_11_-EG_6_-OH**/**30% of HS-(CH_2_)_11_-EG_6_-OCH_2_-COOH, 70% of HS-(CH_2_)_11_-EG_6_-OH**/**30% of HS-(CH_2_)_11_-EG_6_-NH_2_, and 100% of HS-(CH_2_)_11_-EG_6_-OH. The specified amounts of ligands were added to working mixtures with the corresponding pH values: 0.1 M sodium phosphate buffer solution (pH 7.2) if the HS-(CH_2_)_11_-EG_6_-OH ligand was used, 0.1 M sodium phosphate buffer solution (pH 8.0) in the case of the mixture of 70% of HS-(CH_2_)_11_-EG_6_-OH**/**30% of HS-(CH_2_)_11_-EG_6_-OCH_2_-COOH, and 0.1 M sodium phosphate buffer solution (pH 6.6) in the case of 70% of HS-(CH_2_)_11_-EG_6_-OH**/**30% of HS-(CH_2_)_11_-EG_6_-NH_2_. After that, the mixtures were incubated at a temperature of 4 °C for 24 h.

After the incubation, the QDs were purified from unbound excess ligands. For this purpose, the QD solutions were placed into the upper chambers of Amicon Ultra-15 10K filter (Millipore SAS, Molsheim, France), and the filter devices were centrifuged upon addition of 15 mL of 0.1 M sodium phosphate buffer (pH 7.2) in the case of HS-(CH_2_)_11_-EG_6_-OH, 0.1 M sodium phosphate buffer (pH 8.0) in the case of the mixture of 70% of HS-(CH_2_)_11_-EG_6_-OH**/**30% of HS-(CH_2_)_11_-EG_6_-OCH_2_-COOH, and 0.1 M sodium phosphate buffer solution (pH 6.6) in the case of 70% of HS-(CH_2_)_11_-EG_6_-OH**/**30% of HS-(CH_2_)_11_-EG_6_-NH_2_. The centrifugation was performed three times at room temperature at 4000 rpm for 10 min. Then, the preliminarily purified and concentrated QD solutions were purified by gel-filtration chromatography on PD MiniTrap G-25 columns (GE Healthcare, Chicago, Illinois, USA) according to the manufacturer’s protocol. For this purpose, 500 μL of a QD solution was applied onto a column preliminarily equilibrated with a 0.1 M sodium phosphate buffer solution with the corresponding pH. 1 mL of the same buffer solution was used for elution. The fractions containing QDs were collected into a separate test tube. After two cycles of QD purification using gel-filtration chromatography, the solution was passed through Whatman Unstop filters with 100 nm pores. The concentrations of the resultant preparations were estimated for subsequent characterization and further analyses.

### 2.6. Estimation of the Optical Properties, Stability, Sizes, and Charges of the Solubilized Quantum Dots

The QD size (hydrodynamic diameter) was measured in each preparation step of water-soluble QDs using a Malvern Zetasizer Nano ZS dynamic light scattering analyzer (Malvern Instruments Ltd., Worcestershire, UK)). The QD size was measured by analyzing electrophoretic light scattering by means of the same instrument. The size and charge of all QD samples were studied in the concentration range of 0.1–0.2 mg/mL. Each measurement was made at least five times and the results were estimated using the standard statistical methods.

The QD colloidal stability was estimated in two different media at two different incubation temperatures. First, two media were prepared for the experiment: 0.1 M sodium phosphate buffer solution (pH 7.2) and the RPMI-1640 culture medium (Thermo Fisher Scientific, Waltham, MA, USA). QDs were placed into each medium to a concentration of 1 mg/mL. Then, all samples were divided into two groups: samples to be incubated at 37°C in a ThermoMixer C thermal shaker (Eppendorf France SAS, Montesson, France) and those to be incubated at room temperature. The hydrodynamic diameter determined by a Malvern Zetasizer Nano ZS analyzer of dynamic light scattering served as a criterion of QD stability. This value was measured daily for five days.

### 2.7. Estimation of Quantum Dot Cytotoxicity In Vitro

The QD cytotoxicity was analyzed using the SK-BR-3 human breast cancer cell line (ATCC, Manassas, VA, USA) and the WI-38 normal human fibroblast cell line (ATCC, Manassas, VA, USA). Before the experiment, the cells were allowed to thaw for 1–2 min in a water bath at the temperature of 37 °C. After thawing, the cells were transferred to 15 mL test tubes containing 5 mL of RPMI-1640 medium (Thermo Fisher Scientific, Waltham, MA, USA). In order to remove the cryoprotectant, the cell suspension was stirred and centrifuged at 1500 rpm for 5 min, and the supernatant was carefully withdrawn in such a way as to minimize the loss of cells. After that, the cells of both lines were grown in culture flasks containing RPMI-1640 medium supplemented with 10% of fetal calf serum, 2 mM *L*-glutamine, penicillin–streptomycin antibiotic, sodium pyruvate, and RPMI-1640 vitamin solution (Thermo Fisher Scientific, Waltham, MA, USA) for complete growth medium in an incubator at 37 °C in a 5% CO_2_ atmosphere. When the cells had formed a monolayer, they were detached from culture flasks with Versene solution. To do this, the culture medium was removed, the cells were incubated in 2 mL of Versene solution for 2–5 min, 5 mL of complete growth medium was added to the flask, and the cells detached from the flask bottom were collected into a centrifuge test tube. The cell suspension was centrifuged at 1500 rpm for 5 min, the cell pellet was resuspended in 5–10 mL of complete growth medium, and the cells were counted in KOVA™ Glasstic™ Slide (Thermo Fisher Scientific, Waltham, MA, USA) and placed into a fresh culture flask, 5 × 10^5^ cells per flask containing 8 mL of complete growth medium.

The effect of the QDs on the viability of cell cultures was estimated using 3-(4,5-dimethylthiazol-2-yl)-2,5-diphenyl tetrazolium bromide (MTT), a tetrazolium dye, according to supplier’s protocol (Thermo Fisher Scientific, Waltham, MA, USA). In the mitochondria of viable cells, MTT was metabolized to form formazan crystals, which have a purple color. The results of this reaction were estimated photometrically.

In order to prepare the MTT test, 0.5 g of the MTT reagent was dissolved in 100 mL of sodium phosphate buffer solution (pH 7.4) to a final concentration of 5 mg/mL, and the resultant solution was filtered through a filter with 0.22 μm pores and placed into 4 mL test tubes. The dissolved MTT reagent was stored frozen at −20 °C, the necessary amount being thawed before the reaction.

For the MTT test, the cells were detached from the bottom of the culture flask and centrifuged at 1500 rpm for 5 min. The pellet was resuspended in complete RPMI-1640 growth medium, and the cells were counted in a hemocytometer. Then, the cells were cultured in a 96-well flat pate, 7 × 10^3^ cells per well containing 180 μL of the culture medium. The growth medium without cells was placed into the wells at the edge of the plate. The plates were incubated at 37°C in a 5% CO_2_ atmosphere overnight for the cells to attach to the well bottom. After that, QD samples with concentrations from 0.2 mg/mL to 0.781 ng/mL prepared by binary dilution in triplicate were placed into the wells that contained cells and, next, the plates were incubated for 24 or 48 h in an incubator at 37 °C in a 5% CO_2_ atmosphere.

After the cells were incubated in the presence of QDs for the specified period of time, the plates were centrifuged at 1500 rpm for 6 min, the supernatant was carefully withdrawn, and the complete RPMI-1640 growth medium was added to the wells. The centrifugation followed by removal of the supernatant and addition of fresh cell growth medium was performed in order to wash off QDs from the cells and eliminate the optical contribution of QDs during the subsequent measurement of the formazan optical density. Then, 20 μL of the MTT reagent solution was added into each experimental well to a final concentration of 1 mg/mL, and the plates were incubated for 4 h at 7 °C in a 5% CO_2_ atmosphere. During this time, formazan crystals were formed in the wells containing viable cells. The plates were centrifuged at 1500 rpm for 6 min, the supernatant was withdrawn, and 150 μL of DMSO was added into each well to dissolve the formazan crystals. After that, the plates were placed into an incubator for 10 min and then stirred on a shaker to make the formazan solution homogeneous. The optical density of the formazan solution in the wells was measured using a Multiskan EX photometric analyzer of immunoenzyme reactions (Thermo Fisher Scientific, Waltham, MA, USA) at a wavelength of 540 nm. The optical density was directly proportional to the number of live cells. The cytotoxicity (CT) was estimated in percent as

(1) $$CT=\left(1-\frac{ODe}{ODc}\right)\times 100\%$$

where ODc and ODe are the optical densities in the control and experimental wells, respectively.

Finally, the inhibitory concentration of QDs that caused death of 50% of the cells (IC_50_) was calculated.

### 2.8. Fluorescence Microscopy

SK-BR-3 human breast cancer cells were seeded into wells of the borosilicate Nunc™ Lab-Tek II Chambered Coverglass slide (Thermo Fisher Scientific, Waltham, MA, USA), 1 × 10^5^ cells in 200 mL of RPMI-1640 culture medium supplemented with 10% fetal calf serum. After the cells adhered to the substrate, the medium was replaced with a serum-free one, and QDs were added to each well on the slides to a final concentration of IC_10_ under standardized conditions to analyze the QD uptake by the cells. An Axio Observer A1 microscope (Carl Zeiss, Oberkochen, Germany) was used for imaging. The measurements were carried out using an Axiocam 506 camera with an LD A-plan 40×/0.55 lens. An excitation filter with the bandpass of 455–495 nm and an emission filter with a bandpass of 505–555 nm were used for excitation and emission at wavelengths of 495 and 519 nm, respectively. The exposure time was 13.33 ms. The QD accumulation in cells was estimated by the fluorescence intensity with the use of the ImageJ software (Wayne Rasband, Bethesda, MD, USA).

### 2.9. Statistical Treatment

The GraphPad Prizm 6 software (GraphPad Software Inc., San Diego, CA, USA) was used for the statistical treatment of the results.

## 3. Results

### 3.1. Size, Composition, and Optical Properties of Water-Insoluble Qds

**Figure 1:**
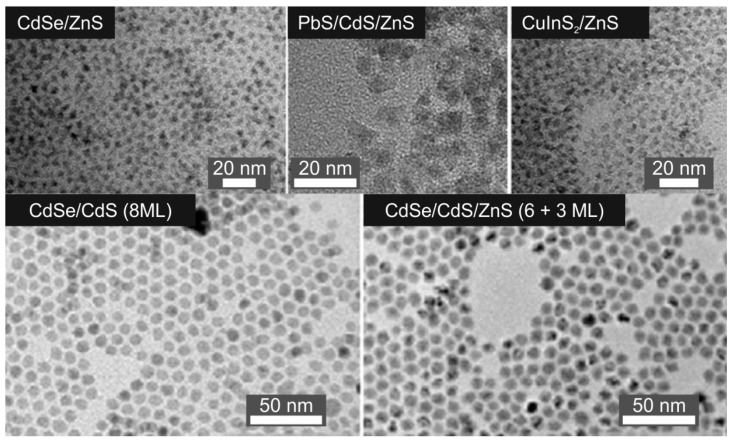
Analysis of the structure of the synthesized quantum dots by means of transmission electron microscopy.

For studying the QD toxicity, we used QDs with different chemical compositions of the cores, which were coated with inorganic shells of different thicknesses to obtain QDs of different sizes. The diameters of the CdSe/ZnS, CdSe/CdS (8 ML), CdSe/CdS/ZnS (6+3 ML), PbS/CdS/ZnS, and CuInS_2_/ZnS QDs were measured from TEM images (Figure 1).

Table 1 shows the chemical compositions, sizes, and optical properties of the water-insoluble QDs. The absorption spectra of the water-insoluble QDs are shown in Figure S1 (Supporting Materials).

### 3.2. Size, Charge, Optical Properties, and Colloidal Stability of Water-Soluble Quantum Dots

**Figure 2:**
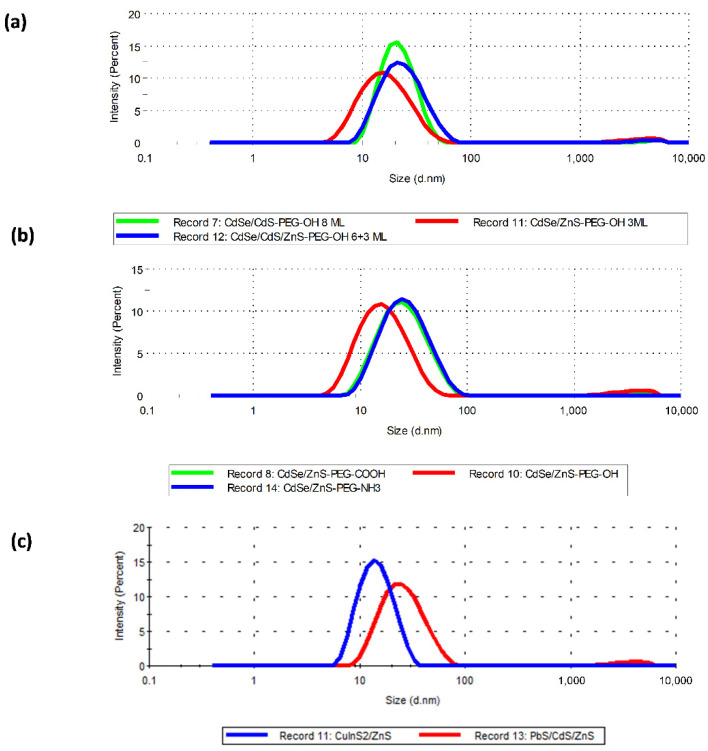
Estimation of the hydrodynamic diameter of quantum dots (QDs) by the dynamic light scattering method: (**a**) QDs with different structures of the inorganic shell; (**b**) QDs with different structures of the organic shell; (**c**) QD-PEG-OH with different cores.

In order to transfer the water-insoluble QDs into the aqueous phase, their surface was modified with DL-cysteine, which was replaced afterwards with thiol-containing PEG derivatives with different end groups to impart different surface charges to the QDs. The sum of the QD diameter and the thickness of the organic shell is not a strictly correct estimate of the size of the QDs modified with PEG derivatives; therefore, below we use the word size to mean the hydrodynamic diameter (HDD) of the water-soluble QDs. The surface charge and HDD were determined, respectively, by the electrophoretic mobility method employing the Doppler effect and by the dynamic light scattering method using a Zetasizer Nano ZS instrument (Figure 2). Table 2 shows the sizes and charges of the QDs used in this study. The number of monolayers (ML) of the inorganic shells applied onto the QD cores are indicated in parentheses. Estimation of the colloidal stability by the dynamic light scattering method showed that the QD HDD remained unchanged for at least five days in a sodium phosphate buffer solution (pH 7.2) or RPMI-1640 culture medium.

We used the *C*dSe/CdS/ZnS (6+3 ML)-PEG-OH and CdSe/CdS (8 ML)-PEG-OH QDs with HDD ≈ 25 nm and CdSe/ZnS-PEG-OH QDs with HDD ≈ 17 nm to study the dependence of QD toxicity on their size. The CdSe/ZnS-PEG-OH, CdSe/ZnS-PEG-COOH, and CdSe/ZnS-PEG-NH_2_ QDs coated with differently charged PEG derivatives and carrying, respectively, low negative, higher negative, and positive surface charges were used to study the dependence of QD toxicity on the charge. The QDs with PbS, CuInS_2_, and CdSe cores, namely PbS/CdS/ZnS-PEG-OH, CuInS_2_/ZnS-PEG-OH, and CdSe/ZnS-PEG-OH QDs, were used to study the dependence of QD toxicity on the chemical composition of their core. Thus, we prepared sets of QDs differing from one another in size, surface charge, and composition. Figure S2 shows the fluorescence spectra of the water-soluble QDs.

### 3.3. In Vitro Cytotoxicity of Quantum Dots

**Figure 3:**
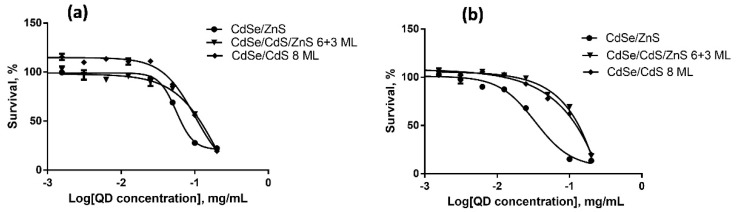
Survival of SK-BR-3 cells upon interaction with CdSe/ZnS-PEG-OH, CdSe/CdS/ZnS (6+3 ML)-PEG-OH, and CdSe/CdS (8 ML)-PEG-OH QDs for (**a**) 24 and (**b**) 48 h as dependent on the QD concentration.

**Figure 4:**
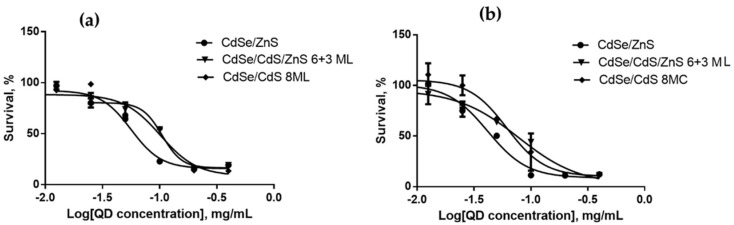
Survival of WI-38 cells upon interaction with CdSe/ZnS-PEG-OH, CdSe/CdS/ZnS (6+3 ML)-PEG-OH, and CdSe/CdS (8 ML)-PEG-OH QDs for (**a**) 24 and (**b**) 48 h as dependent on the QD concentration.

**Figure 5:**
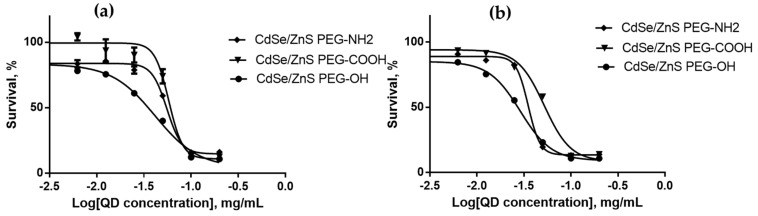
Survival of SK-BR-3 cells upon interaction with CdSe/ZnS-PEG-OH, CdSe/ZnS-PEG-COOH, and CdSe/ZnS-PEG-NH_2_ QDs for (**a**) 24 and (**b**) 48 h as dependent on the QD concentration.

**Figure 6:**
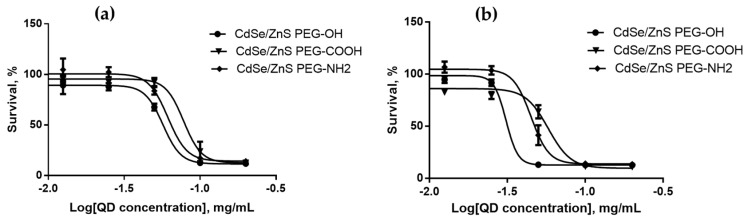
Survival of Wi-38 cells upon interaction with CdSe/ZnS-PEG-OH, CdSe/ZnS-PEG-COOH, and CdSe/ZnS-PEG-NH_2_ QDs for (**a**) 24 and (**b**) 48 h as dependent on the QD concentration.

**Figure 7:**
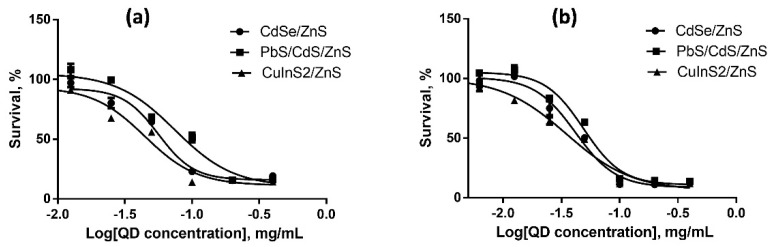
Survival of SK-BR-3 cells upon interaction with CdSe/ZnS, PbS/CdS/ZnS, and CuInS_2_/ZnS QDs for (**a**) 24 and (**b**) 48 h as dependent on the QD concentration.

**Figure 8:**
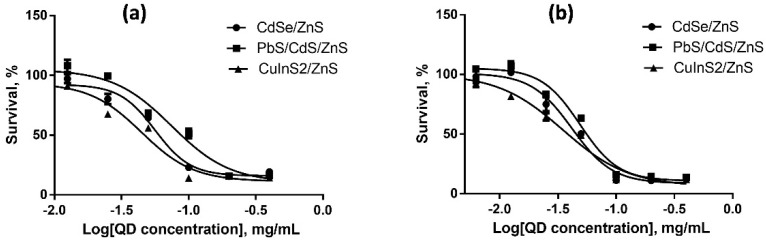
Survival of Wi-38 cells upon interaction with CdSe/ZnS, PbS/CdS/ZnS, and CuInS_2_/ZnS QDs for (**a**) 24 and (**b**) 48 h as dependent on the QD concentration.

We estimated the cytotoxicities of QDs with different HDDs, surface charges, and chemical compositions for SK-BR-3 human breast cancer cells and WI-38 normal human fibroblasts with the use of the MTT test after 24 and 48 h of cell culturing in the presence of QDs. The results of cytotoxicity estimation are shown in Figure 3, Figure 4, Figure 5, Figure 6, Figure 7 and Figure 8 and Table 3.

### 3.4. Interaction of Quantum Dots with Cells

**Figure 9:**
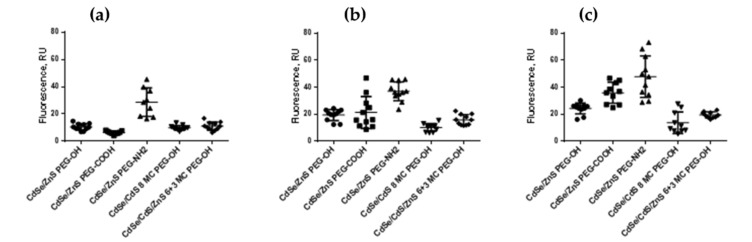
The intensity of fluorescence of SK-BR-3 cells incubated in the presence of QDs with different sizes and charges for (a) 24 h, (b) 48 h, and (c) 72 h.

QDs with identical chemical compositions of the cores and inorganic shells but different HDDs and surface charges were used to analyze the QD penetration into and accumulation in living cells. The CdSe/CdS/ZnS (6+3 ML)-PEG-OH, CdSe/ZnS (8 ML)-PEG-OH, CdSe/ZnS (3 ML)-PEG-OH, CdSe/ZnS (3 ML)-PEG-COOH, and CdSe/ZnS (3 ML)-PEG-NH_2_ QDs were used. The experiments were performed on SK-BR-3 human breast cancer cells because the intracellular transport rate is higher in tumor cells. The cells were examined 24, 48, and 72 h after the QD preparations were added. Figure 9 shows the diagram of QD accumulation in cells based on the calculated integrated fluorescence intensity normalized to the number of cells.

## 4. Discussion

### 4.1. Fabrication and Characterization of Water-Insoluble Quantum Dots

We performed a consistent, systematic analysis to determine the dependence of the toxicity of QDs, spherical fluorescent semiconductor nanocrystals, on their physical and chemical characteristics. For this purpose, we used series of core/shell QDs that differed from one another in the chemical composition of the core (containing cadmium, lead, or copper) and the number of monolayers of the protective inorganic shell of zinc sulfide or cadmium sulfide. By varying the number of shell layers, we fabricated QDs of different sizes, which were used to obtain water-soluble QDs with different HDDs. TEM examination of the synthesized water-insoluble QDs showed that they were highly homogeneous in size.

### 4.2. Obtaining Water-Soluble Quantum Dots

At the first stage of QD solubilization, we used the low-molecular-weight thiol-containing ligand DL-cysteine [17]. This compound allows QDs to be effectively transferred from the organic to the aqueous phase through the ligand substitution reaction of the organic preservatives adsorbed on the QD surface after the synthesis. This ensures colloidal stability of the QD preparations for at least several days, which considerably simplifies the subsequent modification of the QD surface. However, cysteine-coated QDs are prone to spontaneous oxidation and, hence, are stable only in media containing a reducing agent. Because we had to obtain highly stable homogeneous QD preparations with different surface charges, we replaced cysteine with low-molecular-weight thiol-containing PEG derivatives for subsequent modification of the QD surface. PEG-based ligands were used because they are nontoxic and have even been approved by FDA as materials for bone tissue regeneration [19]. PEG is highly biocompatible and nonimmunogenic, and it prevents protein adsorption on QDs [20]. The resultant decrease in the nonspecific interaction of QDs with proteins prolongs their circulation time in blood [21]. In addition, PEG-coated QDs have a high colloidal stability at different pH values due to the interaction of the polyethylene glycol chain with the solvent molecules [22]. This sets them apart from the surface ligands based on organic acids, which are stable only at neutral or acidic pH values [23].

In order to impart different electrical charges to the QD surface, we used the PEG derivatives that had a hydroxyl, a carboxyl, or an amine group at one end and an aliphatic chain with a backbone of 11 carbon atoms and a terminal SH group at the other end: HS-(CH_2_)_11_-EG_6_-OH/COOH/NH_2_ (the SH group serving for the displacement of cysteine molecules from the QD surface). The hydrophobic aliphatic chains of the ligand formed an additional dense shell around the QDs, which allowed them to retain the QD colloidal stability for a long time. This approach made it possible to obtain a series of CdSe/ZnS QDs that differed from one another only in the surface charge: (1) positive (the organic shell consisting of 70% of HS-(CH_2_)_11_-EG_6_-OH and 30% of HS-(CH_2_)_11_-EG_6_-NH_2_), (2) low negative (100% of HS-(CH_2_)_11_-EG_6_-OH), and (3) higher negative (70% of HS-(CH_2_)_11_-EG_6_-OH and 30% of HS-(CH_2_)_11_-EG_6_-OCH_2_-COOH). Hereinafter, the QDs with these organic shells are referred to as CdSe/ZnS-PEG-NH_2_, CdSe/ZnS-PEG-OH, and CdSe/ZnS-PEG-COOH, respectively. The ratio of 7:3 between the neutral hydroxyl end group and the negative carboxyl or positive amine group was used because it ensured colloidal stability of QDs in a wide pH range due to the optimal distribution of charged groups over the QD surface [24] while imparting the desired electrical charge to the surface. All the other types of QDs were modified with the PEG derivative that had a hydroxyl end group (hereinafter referred to as PEG-OH). It should also be noted that the QDs with this organic shell remained colloidally stable in biological media not only for the first 5 days, but also afterwards, as long as a year after synthesis (data not shown). This makes them suitable, e.g., for using in diagnostic kits with a shelf life close to that of the traditional organic fluorescent dyes.

An undoubted advantage of this study is that all experiments were performed with comprehensively characterized QDs, each type of them obtained in a single synthesis, which prevented variations in applying the organic surface ligands [25].

### 4.3. In Vitro Cytotoxicity of Quantum Dots

Study of QD cytotoxicity in in vitro models is simpler and less expensive than experiments on laboratory animals, although it yields less information on the possible risks related to the use of nanomaterials. While it is true that only in vivo models can clarify the issues of the distribution, accumulation, and excretion of QDs, as well as their toxicity for tissues, organs, and systems, estimation of the QD toxicity for cell lines is necessary for initially screening the substances studied and determining whether or not they have a damaging effect on cells.

We used both normal and tumor cell lines (WI-38 human fibroblasts and SK-BR-3 human breast cancer cells). It is known that the transport of substances into tumor cells is more rapid than that into normal cells because of the rapid division and the related high metabolic rate of the former. In addition, the pH inside tumor cells may be different due to a more intense glycolysis [26], which may affect the QD stability and their surface charge because of the protonation and deprotonation of the surface ligands. The surface charges of the membranes of both tumor and normal cells may also differ from each other, because the tumor cell membrane contains more negatively charged lipids. This may also affect the QD interaction with the cells [27].

### 4.4. Dependence of the In Vitro Cytotoxicity of Quantum Dots on Their Hydrodynamic Diameter

Experiments were performed with three types of QDs with the same chemical composition whose surface was functionalized with PEG-OH. CdSe/ZnS-PEG-OH QDs had the smallest HDD (about 17 nm); the HDD of **C**dSe/CdS/ZnS (6+3 ML)-PEG-OH and CdSe/CdS (8 ML)-PEG-OH QDs was about 26 nm. The ζ-potentials of both types of the larger QDs were approximately equal, varying between −9 and −11 mV, and the ζ-potential of the 17 nm QDs was −5 mV. Figure 3 and Figure 4 show the dependence of QD cytotoxicity on their HDD for the SK-BR-3 and WI-38 cell lines, respectively. These data were used to calculate the IC_50_ value for each type of QDs (Table 3).

Estimation of the IC_50_ shows that the smaller CdSe/ZnS-PEG-OH QDs are more toxic than the larger QDs for both cell lines tested. It is known that QDs introduced into a cell culture may interact with the components of the culture medium. The formation of stable complexes of the QDs and proteins of the medium may promote the active (receptor-dependent) or passive transport of the QDs [28]. In this case, the QD size and surface characteristics determine the profile and conformational state of proteins in these complexes, which also affects the effectiveness of their transmembrane and intercellular transport. QDs penetrate into cells via phagocytosis, pinocytosis, and macropinocytosis, whose effectiveness is inversely proportional to the size of the transported particles [14]. Irreversible damage of the membrane by larger QDs penetrating through it is more probable compared to smaller QDs. This explains why tumor cells are more sensitive to large QDs than normal cells are: their more intense metabolism determines an equally more intense transmembrane transport [29], which increases the probability of cell death due to membrane disruptions. At the same time, smaller QDs are more toxic than larger ones for all types of cells, because smaller QDs more readily penetrate into a cell via nonspecific transport mechanisms and cause cell death mainly through oxidation of inner cell components rather than damage of the plasma membrane. The more effective transport of small QDs into cells was further confirmed by our experiments on QD accumulation in cells, which showed that QDs with an HDD of about 17 nm more rapidly penetrate through the cell membrane than larger QDs. In addition, note that cells of all types are more resistant to disruptions of plasma membrane than to oxidative destruction of their inner components. After a longer culturing (48 h), the cytotoxicity of QDs was increased (Figure 3b and Figure 4b) compared to that after a 24 h culturing (Figure 3a and Figure 4a), whereas the difference in the QD cytotoxicity between tumor and normal cells was decreased, which suggests intracellular accumulation of the QD preparations during long-term incubation.

### 4.5. Dependence of the In Vitro Cytotoxicity of Quantum Dots on Their Surface Charge

We studied the effect of the QD surface charge on their cytotoxicity using CdSe/ZnS QDs modified with different ligands: PEG-OH derivatives imparting a low negative charge to the QD surface, a mixture containing PEG-COOH imparting a greater negative charge, and a mixture containing PEG-NH_2_ imparting a positive charge. The QDs with a low and high negative charge had equal HDDs of 15–16 nm, and the HDD of positively charged QDs was 22–23 nm. Figure 5 and Figure 6 show the dependence of the QD cytotoxicity on their surface charge for SK-BR-3 and Wi-38 cells, respectively. We used these data to calculate the IC_50_ for each type of QDs (Table 3).

The QDs with a low negative charge have were to be the most toxic for both cell types, whereas the QDs with a high negative charge are the least toxic. It is obvious that the QD surface charge influences the rate of their transport through the plasma membrane, as well as their interaction with inner cell components. The physical, chemical, and reactive parameters of the surface ligands determining the surface charge also make a considerable contribution to the transport effectiveness [13]. The QD charge influence their toxicity in a highly cell-specific way, because some cells are characterized by an increased sensitivity to positively charged QDs [30], whereas others are more susceptive to the action of negatively charged ones [31]. Our measurements show that QDs with a low negative surface charge are the most toxic for both normal and cancer cells. Positively charged QDs most readily penetrate into cells and accumulate there, but they have no strong toxic effect compared to other QD types, probably, because of molecular interactions with cell components. After a prolonged (48 h) incubation of the cells in the presence of QDs (Figure 5b and Figure 6b), the IC_50_ values for both cell types were proportionally decreased compared to these values received after 24 h of incubation (Figure 5a and Figure 6a). This suggests that precisely the QD transport rate depending on the surface charge is the limiting factor for the expression of toxicity.

### 4.6. Dependence of the In Vitro Cytotoxicity of Quantum Dots on Their Chemical Composition

Dependence of the QD toxicity on their chemical composition was studied in a series of core/shell QDs with cores of different chemical compositions coated with identical ZnS inorganic shells. The surface of all types of QDs was functionalized with PEG-OH. We studied QDs with cores of heavy metal salts (CdS and PbS) and with CuInS_2_ cores. All the QDs had a low negative surface charge varying from −6 to −10 mV, which was within the measurement error. The HDDs of the QDs with Cu- and Cd-containing cores were of about the same size, 16 nm, and that of the QDs with Pb-containing cores was twice as large (32 nm).

Figure 7 and Figure 8 show the data on the dependence of QD cytotoxicity for SK-BR-3 and WI-38 cells, respectively, on the composition of the QD core. The IC_50_ values for different types of QDs calculated from these data are shown in Table 3.

The results show that QDs with equal HDDs but different core compositions have almost equal IC_50_ values for both tested cell lines, although there are published data that QDs with CuInS_2_ cores are less toxic than those with CdSe cores [32]. The most plausible explanation of our data is that the ZnS inorganic shell and the additional shell formed by the aliphatic parts of the ligand molecules effectively prevent QD degradation and, hence, protect cells against the heavy metals of the QD core. This is further confirmed by the absence of a substantial difference in cytotoxicity between the two types of QDs after 24 and 48 h of cell incubation in the presence of these nanomaterials (Figure 7a, Figure 7b and Figure 8a, Figure 8b). The cytotoxicity of the QDs with Pb-containing cores and CdS or ZnS shells after 24 h of incubation was lower than the toxicities of the other two QD types studied, which may have been determined by the substantially larger HDD of the former QDs. After 48 h of incubation, the IC_50_ of the QDs with lead-containing cores for tumor cells became comparable to the IC_50_ of those with cadmium- and copper-containing cores. In contrast, the IC_50_ of the lead-containing QDs for the cells with normal metabolism remained considerably higher compared to the other QD types. These data suggest a more rapid transmembrane transport of lager QDs by tumor cells. We can conclude that the cytotoxicity of QDs of similar sizes but different chemical compositions of the core are almost equal to one another, because the conditions of in vitro cell culturing do not induce QD degradation. Thus, the difference in cytotoxicity between the types of QDs studied is mainly determined by their different sizes.

### 4.7. Interaction of Quantum Dots with Cells In Vitro

For experiments on the QD penetration through the plasma membrane and accumulation in cells, we used QDs with the same chemical composition but different HDDs and surface charges: CdSe/CdS/ZnS (6+3 ML)-PEG-OH, CdSe/CdS (8 ML)-PEG-OH, CdSe/ZnS-PEG-OH, CdSe/ZnS-PEG-COOH, and CdSe/ZnS-PEG-NH_2_. The study was performed on SK-BR-3 human breast cancer cells because tumor cells have a high rate of intracellular transport. The cells were studied 24, 48, and 72 h after the addition of the QDs of different types. Figure 9 shows the diagram of QD accumulation in cells estimated by the integrated fluorescence intensity normalized to the number of cells. As seen from the diagram, positively charged QDs, despite their larger HDD, most effectively penetrated through the cell membrane as early as after 24 h of incubation. This was because the negative charge of the membrane facilitated the penetration of the positively charge QDs. They could also effectively penetrate through the nuclear membrane and interact with the negatively charged sugar–phosphate backbone of DNA. After 48 and 72 h of incubation, the QDs with smaller HDDs penetrated into cells more effectively, which confirms the conclusion that positively charged QDs with a small HDD are the most toxic for cells in vitro.

## 5. Conclusions

A new approach to the optimization of the characteristics of potentially biocompatible fluorescent semiconductor nanocrystals has been used to obtain a series of water-soluble core/shell QDs differing from one another in one of three parameters: chemical composition of the core, size, or surface charge. All the synthesized QDs have an organic outer shell reliably protecting the QD core under the conditions of a cell culture. The physical and chemical parameters of this shell are stable and were comprehensively characterized which, has allowed us to perform systematic analysis of the in vitro cytotoxicity for all types of QDs. The results demonstrate that the smaller the QDs’ size, the higher their in vitro cytotoxicity, with the cytotoxic effect on tumor cells developing more rapidly compared to normal cells. QDs with a low negative surface charge are more cytotoxic than QDs with a greater negative or a positive charge, tumor cells being more susceptible to this effect. In contrast, the chemical composition of the QD core has practically no effect on the QD cytotoxicity in vitro, provided that the epitaxial inorganic shell and the additional outer shell of the modifying ligand (ensuring the colloidal stability and biocompatibility of QDs) reliably protect the QDs from degradation.

## Figures and Tables

**Table 1:** Composition and optical properties of the synthesized water-insoluble quantum dots.

QD Type	λ_max exc._, nm	λ_max fl._, nm	Diameter, nm
PbS/CdS/ZnS	1300	1400	8.9
CuInS_2_/ZnS	550	690	4.5
CdSe/ZnS	570	590	5.5
CdSe/CdS (8 ML)	470	590	8.5
CdSe/CdS/ZnS (6+3 ML)	450	610	9.2

**Table 2:** Sizes and surface charges of the QDs used in this study.

QD Composition	Size, nm	ζ-Potential, mV
PbS/CdS/ZnS-PEG-OH ^1^	32.04 ± 0.87	–10.60 ± 2.92
CuInS_2_/ZnS-PEG-OH	16.08 ± 0.51	–6.12 ± 1.81
CdSe/CdS/ZnS (6+3 ML)-PEG-OH	26.48 ± 0.92	–8.88 ± 1.87
CdSe/CdS (8 ML)-PEG-OH	25.86 ± 1.22	–11.20 ± 1.37
CdSe/ZnS-PEG-OH	16.74 ± 0.28	–4.72 ± 0.38
CdSe/ZnS-PEG-COOH ^2^	15.37 ± 0.14	–17.80 ± 3.01
CdSe/ZnS-PEG-NH_2_^3^	22.77 ± 0.36	6.43 ± 1.12

^1^ PEG-OH denotes 100% of HS-(CH_2_)_11_-EG_6_-OH. ^2^ PEG-COOH denotes a mixture of 70% of HS-(CH_2_)_11_-EG_6_-OH and 30% of HS-(CH_2_)_11_-EG_6_-OCH_2_-COOH. ^3^ PEG-NH_2_ denotes a mixture of 70% of HS-(CH_2_)_11_-EG_6_-OH and 30% of HS-(CH_2_)_11_-EG_6_-NH_2._

**Table 3:** Effects of the parameters of QDs of different types on their IC_50_ for SK-BR-3 human breast cancer cells and WI-38 human fibroblasts.

Time, h	QD Type	SK-BR-3		WI-38	
		IC_50_, mg/mL	SD ^1^	IC_50_, mg/mL	SD ^1^
**Effect of the hydrodynamic diameter**					
24	CdSe/ZnS-PEG-OH	0.044	0.025	0.044	0.005
	CdSe/CdS/ZnS (6+3 ML)-PEG-OH	0.058	0.003	0.108	0.004
	CdSe/CdS (8 ML)-PEG-OH	0.053	0.003	0.104	0.003
48	CdSe/ZnS-PEG-OH	0.031	0.018	0.032	0.008
	CdSe/CdS/ZnS (6+3 ML)-PEG-OH	0.046	0.001	0.056	0.009
	CdSe/CdS (8 ML)-PEG-OH	0.035	0.002	0.046	0.002
**Effect of the surface charge**					
24	CdSe/ZnS-PEG-OH	0.044	0.025	0.044	0.004
	CdSe/ZnS-PEG-COOH	0.058	0.005	0.078	0.003
	CdSe/ZnS-PEG-NH_2_	0.055	0.003	0.061	0.009
48	CdSe/ZnS-PEG-OH	0.031	0.018	0.032	0.008
	CdSe/ZnS-PEG-COOH	0.052	0.003	0.058	0.003
	CdSe/ZnS-PEG-NH_2_	0.035	0.006	0.045	0.009
**Effect of the chemical composition**					
24	CdSe/ZnS-PEG-OH	0.044	0.025	0.044	0.003
	PbS/CdS/ZnS-PEG-OH	0.083	0.003	0.080	0.011
	CuInS_2_/ZnS-PEG-OH	0.045	0.007	0.051	0.006
48	CdSe/ZnS-PEG-OH	0.031	0.018	0.032	0.008
	PbS/CdS/ZnS-PEG-OH	0.036	0.008	0.054	0.006
	CuInS_2_/ZnS-PEG-OH	0.033	0.011	0.033	0.003

^1^ The standard deviation has been calculated from the results of three independent experiments.

## Data Availability

The data that support the findings of this study are available from the corresponding authors, A.S. and I.N., upon reasonable request.

## Supplementary Materials

The following supporting information can be downloaded at https://www.mdpi.com/article/10.3390/nano12162734/s1: Figure S1. Absorption spectra of the as-synthesized water-insoluble quantum dots; Figure S2. Fluorescence spectra of quantum dots modified with polyethylene glycol derivatives.
