# Supplementary material for: Dependence of Quantum Dot Toxicity In Vitro on Their Size, Chemical Composition, and Surface Charge
Source: Nanomaterials (Basel). 2022 Aug 9;12(16):2734. doi: 10.3390/nano12162734 (PMC9416395; doi:10.3390/nano12162734)
Supplement: Supplementary file 1 [file nanomaterials-12-02734-s001.zip › nanomaterials-1850409-supplementary.pdf]

Supplementary Materials

Dependence of Quantum Dot Toxicity In Vitro on Their Size, Chemical Composition, and Surface Charge

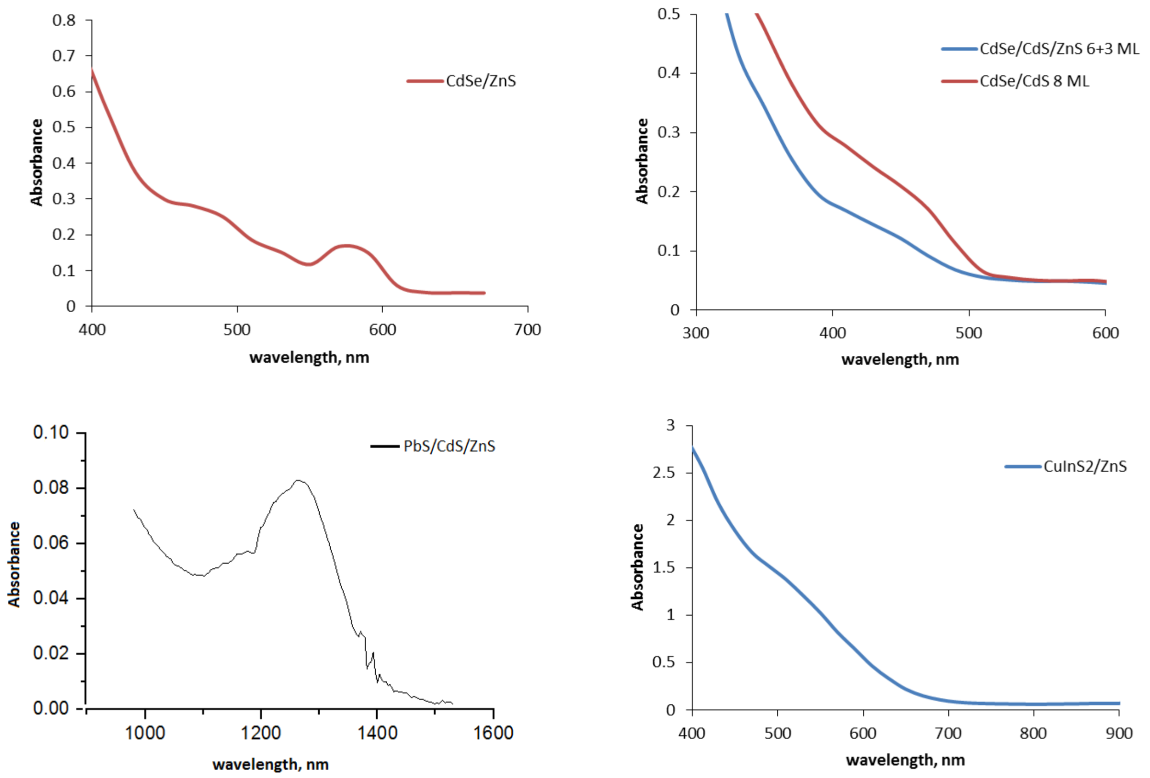

Figure S1. Absorption spectra of the as-synthesized water-insoluble quantum dots.

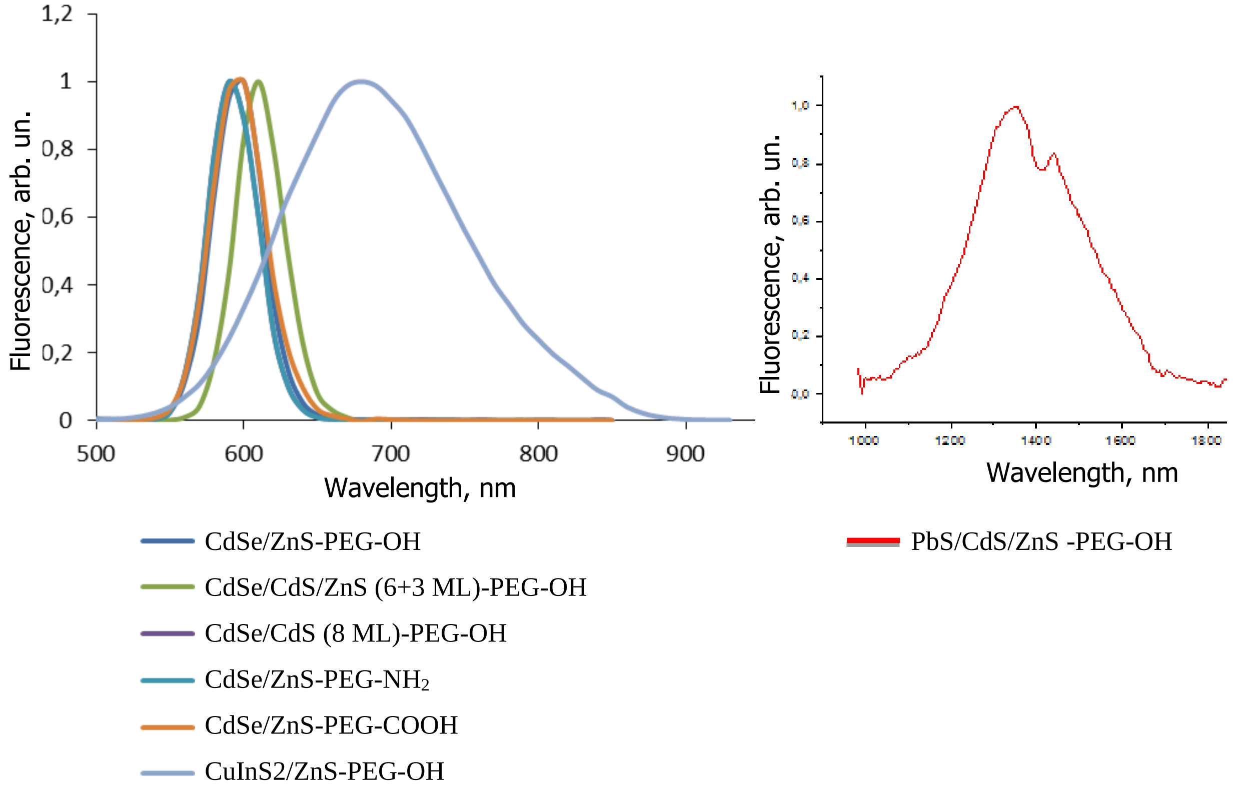

Figure S2. Fluorescence spectra of quantum dots modified with polyethylene glycol derivatives.
